# Paradigm shift: PRRSV-mediated active remodeling of the host immune system—From epigenetic domestication to functional hijacking

**DOI:** 10.3389/fmicb.2025.1738504

**Published:** 2025-12-03

**Authors:** Dianning Duan, Chong Cao, Longxin Qiu, Xiaobing Li, Hongbo Chen

**Affiliations:** 1 College of Life Sciences of Longyan University, Longyan, Fujian, China; 2 Fujian Engineering Research Center for Swine Disease Control and Prevention, Longyan University, Longyan, Fujian, China

**Keywords:** PRRSV, immune remodeling, epigenetics, metabolic reprogramming, functional hijacking, system reset

## 1 Challenging the traditional paradigm of “immunosuppression”

Porcine reproductive and respiratory syndrome virus (PRRSV) is characterized by its immunosuppressive and evasive properties, leading to persistent infection and severe secondary infections in swine herds (Li et al., 2017; Yan et al., 2020). Decades of research have meticulously delineated the classic portrait of PRRSV-mediated immunosuppression: the virus effectively suppresses the production and signaling of Type I interferons (IFN-I; Chen et al., 2016, 2017; Li et al., 2018; Liu et al., 2019; Wei et al., 2020; Liu et al., 2023; Li et al., 2024), impairs the antigen-presenting function of dendritic cells (Wu et al., 2020; Li and Mateu, 2021), and induces lymphocyte apoptosis (Gómez-Laguna et al., 2013; Guo et al., 2018; Maragkakis et al., 2022; Su et al., 2023), primarily through its non-structural proteins (Nsp1, Nsp2, Nsp4, Nsp7, and Nsp11) and structural proteins (nucleocapsid protein). These mechanisms collectively constitute an “immune dysfunction” model, portraying PRRSV as a disruptor that systematically dismantles the porcine antiviral defense system.

However, this conventional model struggles to explain several phenomena: why do pigs exhibit specific, sometimes intense, inflammatory responses amidst seemingly comprehensive immunosuppression (Liu et al., 2010; Xu et al., 2021; Liu J. et al., 2024; Ruedas-Torres et al., 2024)? Why are certain immunosuppressive pathways, such as those involving regulatory T cells (Tregs), paradoxically activated (Cecere et al., 2012; Silva-Campa et al., 2012; Wahyuningtyas et al., 2023)? Emerging research evidence compels a critical re-examination of this fundamental premise. We propose a novel perspective: PRRSV is not a indiscriminate destroyer but rather a shrewd “immune system remodeler.” PRRSV does not simply inactivate the immune system; instead, it actively and precisely reprograms it, aiming to mold the host immune environment into a “novel steady-state” that permits stable viral replication while evading effective clearance. This article systematically elaborates on this disruptive “immune system remodeling” concept through three interconnected core layers: epigenetics, cellular metabolism, and immune function.

## 2 PRRSV utilizes “epigenetic domestication” to achieve persistent immunosuppression

PRRSV induces a contrary, deleterious state of “trained immunosuppression.” This refers to a long-lasting refractory state of the innate immune system, opposite to the enhanced responsiveness seen in “trained immunity,” where an initial challenge with PRRSV epigenetically reprograms innate immune cells (and potentially their progenitors) to mount a weaker response upon subsequent encounters with the same or unrelated pathogens. That is, upon primary contact with PRRSV, the host's innate immune cells are directed toward a long-term state of hypo-responsiveness to the virus and even other pathogens, mediated through virus-induced alterations in chromatin accessibility, histone modifications, and other epigenetic landscapes. PRRSV's alteration of the host immune system is “instructive and persistent,” imprinting a long-lasting “suppressive mark” on innate immune cells via epigenetic modifications. Virus accomplishes this “foundational setting” of host immunity through targeted interventions at two sources: the epitranscriptome and nuclear gene transcription (Wang et al., 2024; Wang Z. H. et al., 2025).

Specific PRRSV proteins may function as “epigenetic writers,” introducing repressive histone marks at the promoter regions of pivotal immune genes (e.g., IFN-β, TNF-α), thereby rendering these genes refractory to activation upon subsequent stimulation (Pang et al., 2023b; Zhang et al., 2024). Recent investigations reveal that viruses (PRRSV, porcine rotavirus, and influenza A virus) hijacks the host m^6^A RNA methylation machinery to promote virus replication (Lin et al., 2023; Wei et al., 2023; Liang et al., 2025; Wang Q. et al., 2025). PRRSV significantly upregulates the methyltransferase METTL3 and facilitates its nucleocytoplasmic translocation. The virus-co-opted METTL3 catalyzes m6A modification of the autophagy receptor SQSTM1/p62 mRNA. This epitranscriptomic modification enhances the interaction between SQSTM1 and the critical IFN pathway kinase IKKε, culminating in IKKε degradation via the selective autophagy pathway, thereby suppressing IFN-I production at a post-translational level (Zhai et al., 2025). This signifies PRRSV's capacity to enforce long-term silencing of innate immune signaling by rewriting the host's “epitranscriptomic code.”

This reprogramming results in a scenario where the host's innate immune system, following the initial viral encounter, exhibits a persistently diminished capacity to respond to subsequent challenges—including PRRSV reinfection or other pathogens—even after the virus is cleared. This epigenetic reprogramming may potentially originate at the level of bone marrow hematopoietic progenitor cells, leading to newly generated myeloid cells universally carrying this suppressive imprint (Wang et al., 2019). This discovery profoundly illustrates PRRSV's capability to subvert the fundamental rules of host cell stress defense, converting antiviral structures into pro-viral niches, serving as a vivid embodiment of cellular function being remodeled by the viral “code” operating at and above the epigenetic level. By rewriting the host's epigenetic and epitranscriptomic information, PRRSV effectively performs “underlying code implantation” into the immune system, establishing the cornerstone for subsequent metabolic and functional remodeling.

## 3 PRRSV implements immunosuppression through “metabolic checkpoints”

PRRSV-induced immunosuppression is deeply rooted in its “hijacking” of immune cell metabolism, effectively dismantling the immune response at its functional foundation by usurping critical nutritional resources. The activation, proliferation, differentiation, and effector functions of all immune cells are intrinsically dependent on specific metabolic pathways (Saravia et al., 2020; Wu et al., 2025). We posit that PRRSV establishes a “metabolic supremacy” by systemically commandeering central metabolic pathways, fundamentally depriving immune cells of their essential “fuel.”

PRRSV potently induces Indoleamine 2,3-dioxygenase-1 (IDO1), leading to rapid depletion of local tryptophan. Tryptophan scarcity directly impedes T cell proliferation, while its metabolite, kynurenine, acts as a potent immunosuppressant (Liu X. et al., 2024; Kim et al., 2025). PRRSV replication necessitates substantial lipid resources; it likely hijacks host lipid metabolism pathways, potentially causing aberrant lipid accumulation in immune cells, inducing lipotoxicity, or disrupting cell membrane fluidity and signal transduction (Ma et al., 2024; Zheng et al., 2024; Xie et al., 2025). PRRSV hijacks host glycolysis and glutamine metabolism, reprogramming energy metabolism to promote virus replication (Pang et al., 2023a; Guang et al., 2024). This perspective reframes immunosuppression from a “signaling pathway malfunction” to a state of “metabolic resource exhaustion,” explaining the frequent inefficacy of simple immune agonists. Metabolic hijacking constitutes the driving layer of the remodeling process. Epigenetic alterations necessitate functional consequences, and metabolic pathways represent the crucial bridge linking gene expression to cellular function. By exerting control over metabolism, PRRSV translates the foundational epigenetic settings into functional paralysis of immune cells, operating at the level of energy and biomass provision.

## 4 PRRSV induces not “immunosuppression” but “immune hijacking”

PRRSV does not merely suppress immunity; it executes “precision regulation,” effectively transforming components of the immune system into accomplices for viral replication. While traditional focus has centered on PRRSV suppression of Th1-type responses like IFN-γ (Cui et al., 2024; Sun et al., 2025), our novel viewpoint emphasizes that PRRSV concurrently selectively activates certain immunoregulatory pathways. For instance, PRRSV infection actively induces the expansion and activation of immunosuppressive Regulatory T Cells (Tregs; Wongyanin et al., 2012; Nedumpun et al., 2018). These “virus-induced Tregs (viTregs)” are not functionally impaired but are rather hyperactive suppressive units that actively inhibit effector T cells and dendritic cells, achieving a state of immune hijacking akin to “using the host's own spear against its shield.”

PRRSV infection of alveolar macrophages serves purposes beyond mere replication. Substantial evidence indicates the virus drives macrophages toward a unique “M2-like” or “tolerogenic-like” phenotype (Wang et al., 2017; Wahyuningtyas et al., 2021; Gong et al., 2023). These polarized macrophages exhibit high expression of IL-10 and Arginase-1, coupled with low expression of MHC-II and co-stimulatory molecules, thereby actively fostering an anti-inflammatory and tolerant microenvironment (Peng et al., 2009; Nedumpun et al., 2019; Martinenaite et al., 2025). This represents not a loss of cellular function, but rather a malicious “reprogramming” of its functional state by the virus. PRRSV extends its hijacking repertoire to fundamental cellular housekeeping functions. The viral non-structural protein NSP4 actively mediates the translocation of the host factor TRIM28 from the nucleus to the cytoplasm, thereby initiating autophagosome formation and converting the cellular waste-disposal mechanism of autophagy into a tool for viral replication (Chen et al., 2025). Furthermore, research revealed that PRRSV nsp1β actively induces and hijacks stress granules, reprogramming them from antiviral platforms into “safe houses” that promote viral replication, via specific interaction with G3BP1 (Gao et al., 2022). These findings underscore the virus's profound capacity to rewrite the basic operational rules of host cell biology.

In addition, investigations have identified that PRRSV infection upregulates the host actin-binding protein (FLNA) and integrin ITGα5, actively remodeling the host cell cytoskeleton to create favorable physical conditions for bacterial adhesion and invasion (Liu et al., 2025). This conclusively demonstrates that PRRSV's remodeling capability extends to the level of cellular physical architecture and the microenvironment, revealing a novel mechanism, independent of classical immunosuppression, wherein the virus acts as an “intracellular environment architect,” directly contributing to secondary infections. Through the precise manipulation of specific immune cell functions and core cellular pathways, PRRSV actively constructs an immunosuppressive microenvironment. All phenomena traditionally categorized under “immunosuppression” are, in fact, the ultimate manifestations of this active hijacking and domestication process.

## 5 Integrated framework and derived novel intervention paradigms

**Figure 1:**
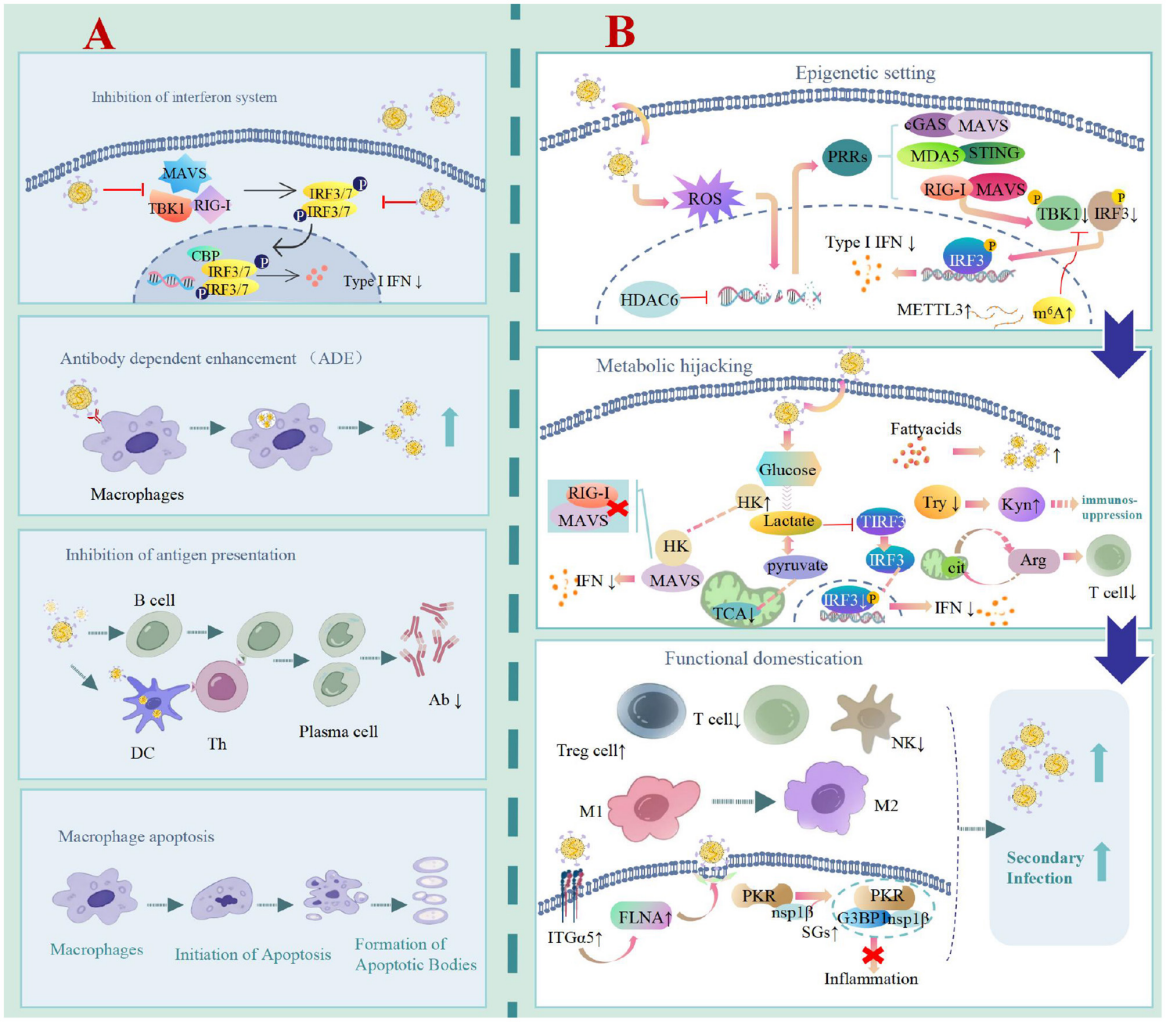
Disruptive viewpoint on PRRSV induced immune suppression mechanism. **(A)** Traditional understanding: PRRSV inhibits IFN response, induces lymphocyte apoptosis, and reduces antigen presentation function, which is a comprehensive “functional shutdown.” **(B)** Disruptive viewpoint: PRRSV is not blind destruction, but rather precise regulation. Try, tryptophan; Kyn, kynurenine; Arg, arginine; cit, citric acid; SGs, stress granules; P, phosphate.

The aforementioned three perspectives are not isolated but constitute a coherent, multi-layered viral pathogenic strategy (Figure 1). PRRSV initially performs a “foundational setting” via epigenetic domestication, achieving long-term reprogramming of the host innate immune system. Building upon this foundation, it establishes local metabolic checkpoints during infection, depriving adaptive immune cells of their response capacity. Concurrently, it actively hijacks the immune regulatory network (e.g., inducing Tregs, polarizing macrophages) and subverts core cellular functions to construct a proactively maintained, virus-favoring immunosuppressive microenvironment. The clinically observed “immunosuppression” is precisely the final phenotypic manifestation of this active, precise, multi-layered remodeling process.

Based on this disruptive framework, our control strategies must undergo a fundamental transformation: shifting from attempting to “enhance” an immune system that has been profoundly remodeled by the virus and resides in an aberrant steady state, toward developing strategies focused on “releasing the hijack” and “resetting homeostasis.” Derived intervention paradigms include: Epigenetic and Stress Response Reset: Exploring the use of low-dose epigenetic drugs or developing small-molecule inhibitors targeting critical virus-host interfaces during specific temporal windows of infection, aiming to “erase” the virally imposed suppressive imprint and restore the cell's autonomous antiviral programming. Metabolic Microenvironment Repair: Designing “metabolic cocktail therapies” incorporating IDO inhibitors and supplements of critical amino acids, aimed at restoring the metabolic fitness of immune cells and providing the essential underlying energetic support for their functional execution. Immune Checkpoint Blockade and Cellular Function Reversal: Drawing inspiration from successes in cancer immunotherapy and autoimmunity, exploring the short-term, localized application of Treg function inhibitors or immune checkpoint blockers to disrupt the immune tolerance established by the virus, concurrently developing immunomodulators capable of reversing the “M2-like” macrophage phenotype back to an “M1-like” state.

## 6 Conclusion and perspectives

We propose herein a fundamental paradigm shift: the pathogenicity of PRRSV stems from its active, multi-dimensional, and precise remodeling of the host immune system, rather than passive functional suppression. This “Immune System Remodeling” model integrates recent discoveries across the epigenetic, metabolic, and functional levels, providing a more holistic and profound framework for understanding PRRSV persistence and immune evasion. While this model necessitates further experimental validation, it unequivocally opens new vistas for future research endeavors—such as developing epigenetic pharmaceuticals, metabolic modulators, and combination immunotherapies. Ultimately, devising strategies that circumvent the “rules of the game” established by PRRSV is paramount for uncovering novel pathways to conquer this formidable pathogen.
